# Reimagining Remote Care: An Online Feasibility Study of Telehealth Communication Mediums for Older Adults

**DOI:** 10.1093/geroni/igaf122.1902

**Published:** 2025-12-31

**Authors:** Renato Ferreira, Leitao Azevedo, George Mois

**Affiliations:** University of Georgia, Athens, Georgia, United States; University of Georgia, Athens, Georgia, United States; University of Georgia, Athens, Georgia, United States

## Abstract

With the growing integration of artificial intelligence (AI) in healthcare, it remains unclear which communication system best enhances comprehension, emotional engagement, and health outcomes for older adults. While audio-based systems like Voice User Interfaces (VUIs) offer accessible and cost-effective solutions, more advanced modalities — such as interactive data visualizations, conversational agents (CAs), and social robots — may provide additional benefits to remote care by incorporating gestures, emotional cues, and interactive feedback. In this presentation, we will share findings from an initial online feasibility study examining older adults’ preferences and responses to various communication mediums when receiving clinical test results and medication instructions. Our study aims to (1) identify which communication medium yields the most favorable outcomes by assessing whether these systems should function solely as informational tools or also as relational agents mimicking healthcare provider interactions, and (2) evaluate how these systems impact comprehension, emotional responses, and risk perception compared to traditional text-based formats. Findings will inform the development of user-centered telehealth solutions that improve health communication for aging populations, optimizing accessibility and effectiveness in remote care settings.

